# Essential Anatomy for the Abdominal Wall Surgeon: Expert Consensus of Anatomical Concepts and Operative Steps for Posterior Component Separation

**DOI:** 10.1002/wjs.70155

**Published:** 2025-11-20

**Authors:** Lawrence Nip, Samuel G. Parker, Andrea Scala, Steve Halligan, Timothy A. Rockall, Rhys Thomas, Pasquale Giordano, Gregory Simpson, Praminthra Chitsabesan, Marja Boermeester, Sarah Zhao, Alastair C. J. Windsor, Miguel Angel Garcia‐Urena

**Affiliations:** ^1^ The Abdominal Wall Unit Croydon University Hospital London UK; ^2^ Centre for Medical Imaging Division of Medicine University College London The Rayne Institute London UK; ^3^ Department of Surgery Royal Surrey County Hospital Guildford UK; ^4^ Minimal Access Therapy Training Unit University of Surrey Guildford UK; ^5^ Department of Surgery Royal London Hospital London UK; ^6^ Department of Surgery Wirral University Teaching Hospital Birkenhead UK; ^7^ York Abdominal Wall Unit York and Scarborough Teaching Hospitals NHS Foundation Trust York UK; ^8^ Department of Surgery Amsterdam University Medical Centres University of Amsterdam Amsterdam Gastroenterology, Endocrinology & Metabolism Amsterdam the Netherlands; ^9^ The Princess Grace Hospital HCA Healthcare London UK; ^10^ Grupo de Investigación de Pared Abdominal Compleja Facultad de Medicina de la Universidad Francisco de Vitoria Hospital Universitario del Henares Madrid Spain

## Abstract

**Introduction:**

Abdominal wall surgery is emerging as a new subspecialty with reconstructive operations becoming increasingly complex. Central to any surgical subspecialty is comprehensive anatomical knowledge, which can be enhanced by cadaver dissection. An expert panel convened to develop a consensus framework highlighting key anatomical concepts and operative steps for teaching posterior component separation.

**Methods:**

The panel consisted of opinion leading abdominal wall surgeons from the UK and Europe. Intellectual content derived from anatomy lectures, training videos, and cadaver dissection instructions formed the basis of the consensus framework. This framework was subsequently implemented during a pilot cadaveric workshop. Afterward, content from the workshop was further refined, resulting in this educational article, endorsed by all authors. This article comprises two sections, (1) theoretical aspects of abdominal wall anatomy; (2) stepwise technical guidance for cadaver dissection.

**Results:**

In the first section, “Essential Anatomy,” we discuss: Anterior abdominal wall musculature, posterior abdominal wall, the semilunar line, preperitoneal space, subxiphoid anatomy and pelvic anatomy. In the second section, “Practical Anatomy taught via Cadaver Dissection,” we discuss: Rives‐Stoppa dissection, caudal extension, cranial extension, classic top‐down transversus abdominis release and posterior component separation with Madrid modification (bottom‐up or “Madrid PCS”).

**Conclusions:**

Using content delivered by senior members of the abdominal wall reconstruction community, this article provides a structured educational framework for teaching posterior component separation. This is intended as a reference guide for surgical training and details the essential anatomical and operative concepts every abdominal wall surgeon should know.

## Graphical abstract

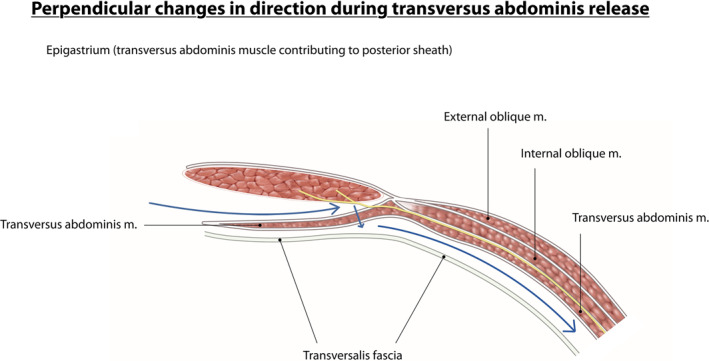

## 1 Background

Abdominal wall reconstruction is becoming a subspecialty in its own right [1, 2]. Recent advances have seen mainstream adoption of new surgical techniques, such as the transversus abdominis release (TAR) [3]. A TAR can be performed either from the top‐down (classic TAR) or by using the Madrid modification (Madrid PCS) from bottom‐up [4]. These are challenging to perform and require detailed anatomical knowledge. For clarity, the terms “TAR” and “posterior component separation” can be used interchangeably and refer to the same surgical procedure. Despite the growing complexity of ventral hernia repair, modern training curricula omit such aspects [5]. Consequently, the pathway to competency often depends on local mentorship opportunities which can be variable and lack standardization [5]. In response to this educational gap, the authors convened to develop a consensus for teaching posterior component separation (PCS) with a focus on anatomy and operative “pitfalls.” The authors propose that this article details the essential anatomical and operative concepts every abdominal wall surgeon should know and can prepare attendees of subsequent cadaveric courses.

## 2 Methods

This work represents a structured consensus process involving a group of key opinion leading UK and European abdominal wall surgeons with substantial experience in abdominal wall reconstruction. All participating members have large abdominal wall reconstruction practices, have longstanding involvement with anatomic and cadaveric teaching, and/or have published widely in the abdominal wall literature. The development process followed principles of consensus development methods (CDMs) [6] which involve repetitive interactions of a working group to reach general agreement. While a formal Nominal Group Technique was not used, our approach aligned with the core elements of iterative expert consultation and structured discussion.

Initially, the group met virtually (Microsoft Teams, Microsoft Corporation, United States) over a 6‐month period to generate ideas for an educational framework for teaching PCS. Drawing on established educational practices, the panel considered theoretical content combined with cadaver dissection to be the most valuable platforms for disseminating understanding [7, 8]. Teaching materials used for cadaveric training such as PowerPoint slides, training videos and instructions for dissection were independently collated and then collectively reviewed. Once agreement was reached on themes for inclusion, the resources underwent content analysis (by L.N. and S.G.P.) to identify core anatomy and operative steps which formed the basis for development of a pilot cadaveric workshop.

The pilot cadaveric workshop was delivered on 12th and 13th March 2025 at the Minimal Access Therapy Unit (MATTU), Royal Surrey County Hospital, Guildford, United Kingdom. Fresh frozen cadavers were obtained for the workshop from the University of Surrey. Attendees were young consultants and senior specialist trainees within 2 years of training completion. Delegates were selected by expressing a strong interest to subspecialize and develop a consultant practice in abdominal wall reconstruction (AWR). Day 1 focused on abdominal wall anatomy and theoretical aspects of abdominal wall reconstruction with the following themes: mesh placement nomenclature, abdominal wall musculature, semilunar line, preperitoneal space, subxiphoid anatomy, Cave of Retzius and Space of Bogros. Day 2 focused on cadaver dissection, following a similar format to the step‐by‐step dissection protocol proposed by Kulkarni et al. [9]: Rives‐Stoppa dissection, caudal extension, cranial extension, classic top‐down TAR and the bottom‐up Madrid PCS.

After the workshop, content for inclusion in this article was refined before being reviewed by our expert panel. The resulting structured educational framework is presented in two parts serving as a guide for future cadaveric workshops that wish to adopt a similar design: (1) abdominal wall anatomy, organized to highlight structures of relevance to PCS, and (2) stepwise technical guidance for cadaver dissection. Standardized illustrations and representative dissection images taken during the workshop accompany the text so that readers can visualize abdominal wall structures and their relationships. The expert panel were all faculty members of the cadaver workshop and have formally endorsed the final anatomical concepts, technical steps, computerized illustrations, cadaveric photographs and referenced material in this article.

## 3 Essential Anatomy

The myofascial complex of the anterior abdominal wall is of considerable interest to the complex hernia surgeon, and over the last few decades numerous reconstructive techniques have been developed, aiming to restore anatomy by muscle medialization and midline closure [10]. The complex consists of five paired muscles, with associated aponeuroses and surrounding fascial sheaths. Anatomically, they are divided into midline and lateral groups. The midline group comprises the rectus abdominis and pyramidalis, and the lateral group comprises the external oblique, internal oblique, and transversus abdominis. These groups present several potential layers for mesh placement, 10 of which have been named in the International Classification of Abdominal Wall Planes (ICAP) [11]. The most widely used and preferred planes for ventral hernia repair are the retrorectus and retromuscular planes [12]. However, others may be suitable [13] depending on patient and technical factors such as rectus muscle quality, location of previously used mesh and availability of dissection planes.

### 3.1 Abdominal Wall Musculature

Abdominal wall surgeons are expected to be familiar with the origins, insertions and muscle fiber directions of rectus abdominis, external oblique, internal oblique, and transversus abdominis. These can be found in most contemporary anatomical textbooks, and whilst we acknowledge they should be discussed in any comprehensive teaching program on posterior component separation, they are of secondary relevance compared to other anatomical concepts, hence not discussed here.

#### 3.1.1 Anterior and Posterior Rectus Sheath

**FIGURE 1:**
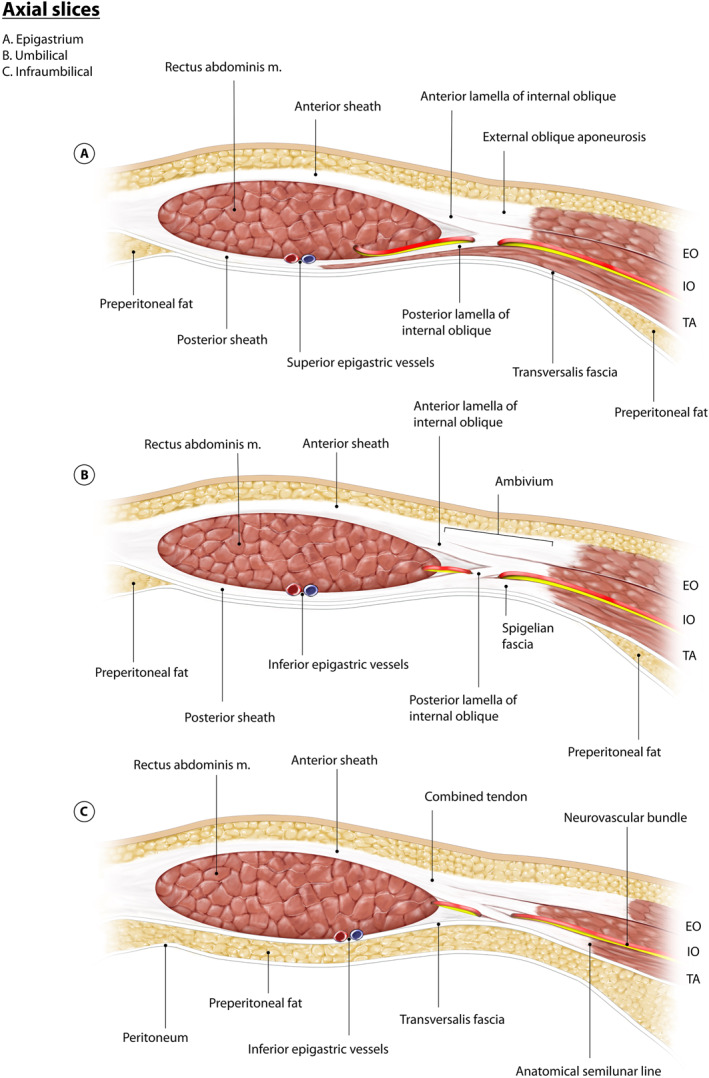
Computerized illustration depicting axial slices at the level of the (A) epigastrium, (B) umbilicus, and (C) infraumbilical region. Note the attachment of transversus abdominis to posterior sheath is muscular in the epigastrium and progressively becomes aponeurotic caudally and note the distribution of preperitoneal fat at each level. The neurovascular bundles in the epigastrium also extend more medial relative to those at the umbilicus and infraumbilical region. EO = external oblique, IO = internal oblique, TA = transversus abdominis.

The anterior and posterior rectus sheaths envelope rectus abdominis and are formed from the aponeurotic contributions of external oblique, internal oblique and transversus abdominis. Specifically, anterior rectus sheath is formed from fibers of external oblique aponeurosis fusing with fibers of anterior lamellae of internal oblique, whilst posterior rectus sheath is formed from fibers of transversus abdominis aponeurosis fusing with fibers of posterior lamellae of internal oblique. Above the arcuate line, this aponeurotic union exhibits a “W” shaped configuration (Figure 1).

**FIGURE 2:**
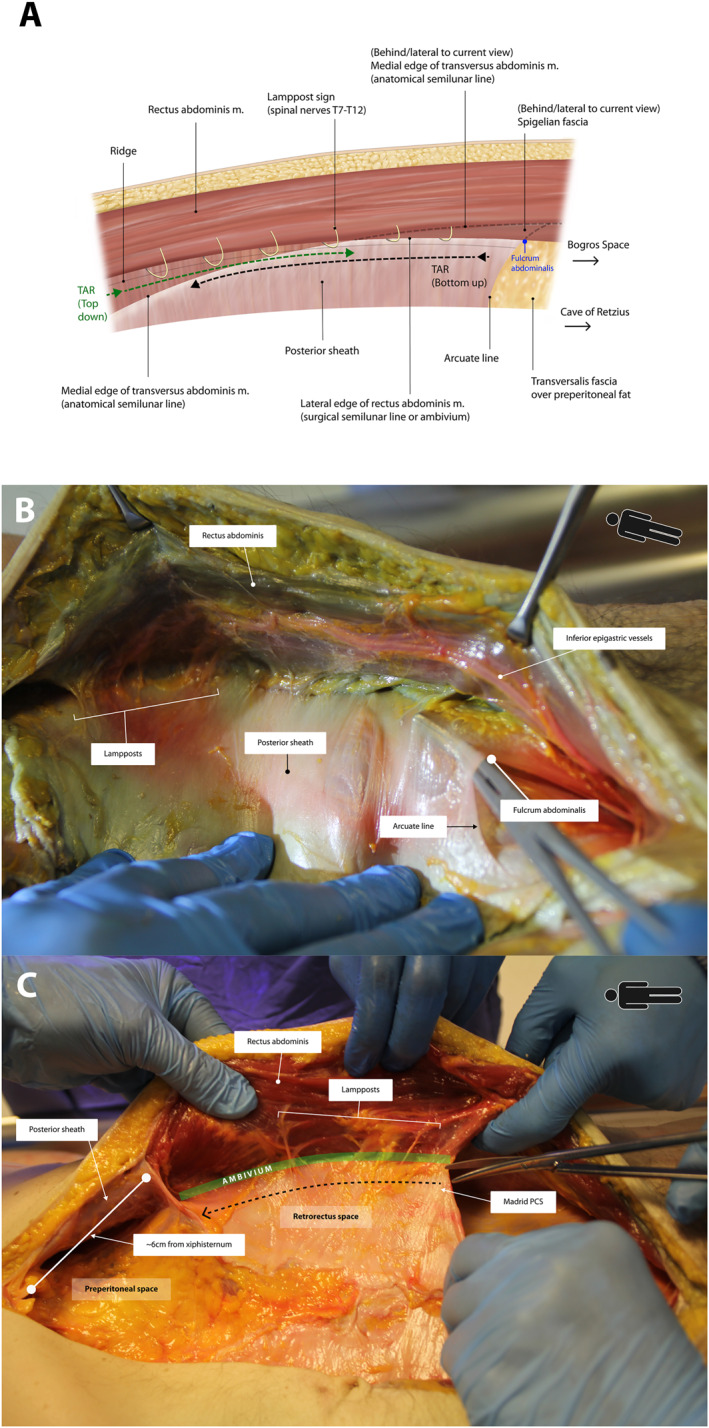
(A) Computerized illustration depicting left sided retrorectus dissection that goes no further than the neurovascular bundles and ridge. Bold arrows show the direction of travel for classic top‐down and bottom‐up (Madrid modification) TAR. Faint dashed lines show the outline of the anatomical semilunar line behind rectus abdominis. (B) Cadaveric photograph after retrorectus dissection. A hemostatic clamp placed underneath the arcuate line shows where bottom‐up TAR commences. (C) Cadaveric photograph demonstrating Madrid PCS. Note that the line of dissection must be medial to the lampposts and ambivium. Pre‐peritoneal dissection should be commenced 6 cm from the xiphisternum to preserve the epigastric neurovascular bundles and fibers of the pars abdominalis.

Cranially, the contribution of transversus abdominis to the posterior rectus sheath is muscular. However, in the lower abdomen its contributions are aponeurotic since the medial limit of transversus abdominis muscle moves in an inferolateral direction (also known as the linea semilunaris) (Figure 1). The arcuate line demarcates the inferior extent of the posterior sheath (Figure 2), and is usually just inferior to umbilical level. Above this line, the rectus abdominis is enveloped by the anterior and posterior rectus sheaths, and below it, the aponeuroses from all three lateral abdominal wall muscles run anterior to rectus abdominis, forming just the anterior sheath. Below the arcuate line, only the transversalis fascia is in direct contact with rectus abdominis posteriorly, with preperitoneal fat deep to this.

#### 3.1.2 Nerve Supply

The lateral abdominal wall muscles are supplied segmentally by the 7th to 11th intercostal and subcostal nerves. Importantly, the nerves and their accompanying vascular supply run in the plane between internal oblique and transversus abdominis before dividing. Lateral cutaneous branches pierce internal oblique to supply external oblique, and anterior cutaneous branches pierce the posterior lamella of internal oblique before supplying the rectus abdominis and anterior abdominal wall skin. The inferior intercostal nerves enter rectus abdominis at its lateral border, but the superior intercostal nerves travel medially on posterior rectus sheath surface for 1–5 cm before entering the undersurface of rectus abdominis [14].

#### 3.1.3 Posterior Abdominal Wall

The structural outline of the posterior abdominal wall is provided by the 11th and 12th ribs superiorly, and the iliac crest inferiorly. Its prime muscles are quadratus lumborum and iliopsoas. These are attached to the axial spine, ribs and pelvic girdle, providing truncal stability. Notably, the vertebral column lies relatively anterior, abutting the retroperitoneum, which creates a concavity to the posterior abdominal wall likened to the shape of a “taco” [15]. This has implications for mesh placement following repair of flank hernias or extreme lateral dissection during TAR.

### 3.2 Semilunar Line

Surgeons must be able to differentiate between “surgical” and “anatomical” (true) semilunar lines. It has become increasingly common for surgeons to incorrectly label the lateral edge of rectus abdominis as the semilunar line, which was an unnamed structure until recently. Coined in a publication by Vierstraete et al. [16], “EIT ambivium” is the correct term, and refers to the surgical semilunar line, or the lateral border of rectus abdominis at which the aponeuroses of external oblique, internal oblique and transversus abdominis converge (Figures 1 and 2). Because this is a topic being debated currently, we propose that the convergence of the aponeuroses at the lateral border of rectus abdominis are simply referred to as the “ambivium”; we will use this term in this manuscript. In reality, the ambivium is not a uniform craniocaudal line of aponeurotic tissue, but has a “W” configuration as mentioned above.

The anatomical semilunar line or linea semilunaris is a separate entity that describes a line of demarcation between muscle and aponeurosis in transversus abdominis, and exhibits a “half‐moon” appearance. With an accurate description of the anatomical semilunar line, two other important surgical landmarks can be delineated [16]; the Spigelian fascia refers to the aponeurosis of transversus abdominis between the anatomical semilunar line and the ambivium (defects in this fascia cause spigelian hernia), and the fulcrum abdominalis describes the junction between the ambivium and the arcuate line (Figure 2A,B).

### 3.3 Pre‐Peritoneal Space

The preperitoneal space lies between transversalis fascia and peritoneum, and contains a thin layer of adipose tissue known as preperitoneal fat. Garcia‐Urena et al. investigated the distribution of preperitoneal fat in cadavers and found that distribution resembled the shape of a trident [17]. This “fatty trident” comprises a root, a midline prong, and left and right lateral prongs, which overlie the paracolic gutters and are in continuity with pararenal and retroperitoneal fat. The cranial aspect of the midline prong is termed the “fatty rhomboid” and is in continuity with the hepatic falciform ligament. Near the apex of the fatty rhomboid and surrounding the xiphisternum is a peri‐xiphoid ball of fat also known as the “fatty triangle” as originally described by Conze [18]. We now know that the “fatty triangle” is part of the larger preperitoneal “fatty rhomboid.”

### 3.4 Subxiphoid Anatomy

**FIGURE 3:**
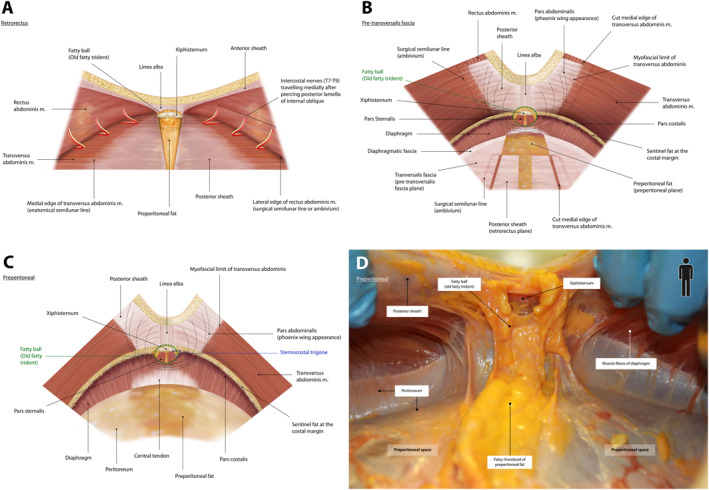
Computerized illustrations and cadaveric photograph of the epigastrium with a cranial view into the subxiphoid region. (A) Dissection in the retrorectus plane has continued cranially up to the “fatty ball.” (B) TAR has been performed; the plane is preperitoneal in the midline and pre‐transversalis laterally. The band of tissue that separates these planes have been cut, revealing a single plane for retromuscular mesh placement. (C) A true preperitoneal view is depicted. The perixiphoid fat (fatty ball) has been pushed up (anterior) and the fatty rhomboid has been pushed down (posterior). (D) Cadaveric photograph showing dissection in a true preperitoneal plane. Peri‐xiphoid fat is pushed anteriorly, and fatty rhomboid is pushed posteriorly. Further dissection cranially would reveal central tendon of the diaphragm.

Like the semilunar line, anatomy of the subxiphoid space is generally poorly understood, probably because primary herniae in this region (M1 based on EHS guidelines [19]) are rare combined with the historical view that mesh overlap behind the costal margin and xiphisternum is impossible. To understand subxiphoid anatomy, it is important to recognize that sub‐diaphragmatic tissue planes correspond with respective abdominal wall planes caudally. Namely, the diaphragmatic fascia is continuous with the transversalis fascia, and the preperitoneal space beneath the diaphragmatic dome is continuous with the preperitoneal space of the anterior abdominal wall. Conversely, the retrorectus plane is not continuous with any corresponding sub‐diaphragmatic plane. This is because the entire rectus sheath complex (anterior and posterior) inserts into the anterior surface of the xiphoid process and costal margin (Figure 3A). Therefore, continuation of the retrorectus plane cranially results in a plane that is superficial (i.e., anterior) to the costal margin, rather than deep to the diaphragmatic dome.

Transversus abdominis has an intimate relationship with the diaphragm via interdigitating muscle fibers on the undersurface of the costal margin. Three distinct muscles arise from the inferior aspect of the diaphragm to insert into the costal margin and fuse with the transversus abdominis, namely pars costalis, pars sternalis, and pars abdominalis (Figure 3B,C). The paramedian and lateral fibers that interdigitate with fibers of transversus abdominis along the undersurface of the costal margin are called the pars costalis. Here, a “sentinel fat pad” [20] overlies the costal margin and is a relatively constant landmark. The most medial diaphragmatic fibers bilaterally, insert onto the posterior surface of the sternum and xiphisternum, termed pars sternalis. Between the transversus abdominis, pars costalis and pars sternalis is a naturally occurring anatomical defect named the sternocostal trigone, through which an eponymous Morgagni hernia can occur (Figure 3C). Moreover, small branches of the internal thoracic artery, termed ensiform vessels, pierce the sternocostal trigones to supply the peri‐xiphoid fat. Unlike pars sternalis and pars costalis, pars abdominalis is relatively recently described. David Lourie [21] coined this term to represent a third set of muscle fibers arising from the diaphragm that insert onto the undersurface of the posterior rectus sheath. Fibers of the pars abdominalis can be recognized by their “phoenix wing” strands, fanning out from the diaphragm on both sides of the midline (Figure 3).

### 3.5 Pelvic Anatomy

#### 3.5.1 Cave of Retzius and Space of Bogros

**FIGURE 4:**
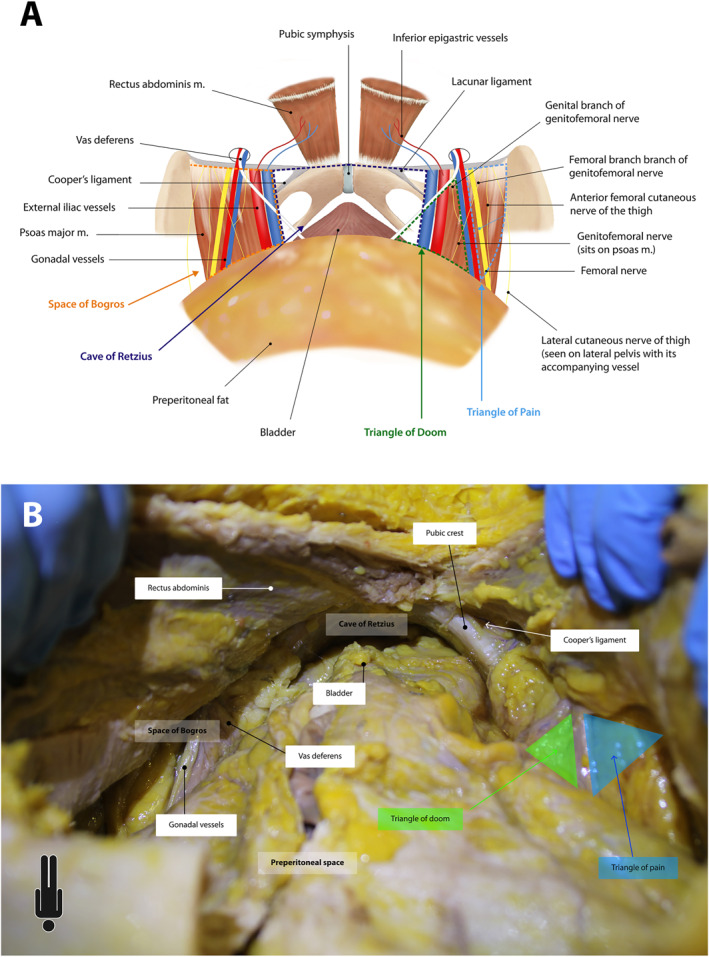
(A) Computerized illustration of the pelvis in the preperitoneal plane. Dashed lines show the Space of Bogros (orange), Cave of Retzius (purple), Triangle of Doom (green) and Triangle of Pain (blue). Note the structures around the myopectineal orifice and their relationships. (B) Cadaveric photograph of the pelvis with a view into the Cave of Retzius and Space of Bogros. The structures of the myopectineal orifice are visible with their corresponding triangles.

The Cave of Retzius is the extraperitoneal retropubic prevesical space [22]. It is bounded anteriorly by pubic bone, posteriorly by urinary bladder, superiorly by peritoneum, inferiorly by pelvic floor musculature, and laterally by inferior epigastric arteries (Figure 4). However, the shape of this space is variable, and defined by the patient's body habitus since it is a potential space rather than a defined anatomical cavity.

The Space of Bogros is often thought of as the lateral extension of the Cave of Retzius. However, the two spaces do not communicate completely freely and there is always a thin layer of connective tissue that separate them. Specifically, the Space of Bogros is the extraperitoneal retroinguinal space, a triangular region situated between the iliac fascia, transversalis fascia and parietal peritoneum [23]. It is bounded anteriorly by transversalis fascia, posteriorly by parietal peritoneum overlying the psoas and external iliac vessels, medially by the inferior epigastric vessels, and laterally by the pelvic sidewalls. The structures of the myopectineal orifice pass through (Figure 4).

#### 3.5.2 Myopectineal Orifice

The myopectineal orifice is a complex network of neurovascular and gonadal structures, and their associated myofascial rings, through which they travel to reach the inguinal canal [24]. Although complex, these structures are easily recognized by their “inverted Y” shape [25]. The iliopubic tract divides this into five distinct anatomical zones [25]. Above the tract lies Hesselbach's triangle medially and the deep inguinal ring laterally, which are zones associated with direct and indirect inguinal hernias. Below the tract from medial to lateral, is the space for femoral hernia, the “triangle of doom,” and the “triangle of pain” (Figure 4). These areas are named after their structures and the potential for iatrogenic injury.

## 4 Practical Anatomy and Instructions for Cadaver Dissection

Attendees practiced midline laparotomy without peritoneal breach, Rives‐Stoppa dissection, caudal extension to the Cave of Retzius and Space of Bogros, cranial extension to the subxiphoid space and diaphragmatic dome, posterior component separation (PCS) via TAR (classical top‐down TAR and Madrid PCS (bottom‐up) on contralateral sides), and mesh placement into the retromuscular plane after TAR.

### 4.1 Rives‐Stoppa

Cadaver dissection began with a generous midline incision to optimize view of subsequent dissection. Careful division of the linea alba reveals midline preperitoneal fat. If possible, the peritoneum should remain intact and approximately 2 cm of preperitoneal fat dissected off the linea and posterior sheath bilaterally to access the medial edge of the posterior sheath. Following this, the posterior sheath is opened close to the midline, thereby exposing the retrorectus space. This plane can be followed laterally until reaching the ambivium, where upward deflection of the neurovascular bundles innervating rectus abdominis is seen as the “lamppost sign” (Figure 2). A common mistake is overzealous lateral sharp extension, risking the neurovascular bundles. If a “ridge” of ligament is seen, this represents the everted anterior lamella of internal oblique and means dissection is too extensive. TAR should only occur medial to the neurovascular bundles and, by default, medial to this ridge (Figure 2A). Lateral dissection beyond the ridge and through the ambivium can cause iatrogenic defects that are notoriously challenging to repair. Bilateral full‐thickness defects (through external oblique aponeurosis) result in the so‐called “mickey mouse hernia” which has a bilobed appearance when viewed in axial section. If external oblique aponeurosis is not fully breached, alternative lateral planes of entry include the retro‐oblique plane between internal oblique and transversus abdominis, as described by Carbonell [26], or the inter‐oblique plane between external oblique and internal oblique, as described by Stumpf [27]. Both injure the neurovascular bundles and are not used in routine clinical practice. Retrorectus dissection is relatively straightforward if the plane has not been utilized previously. However, if so, dissection can be difficult, and patience is required to carefully divide fibrosis subjacent to rectus abdominis.

### 4.2 Caudal Extension

After lateral dissection is complete, caudal extension is commenced, aiming to reach the arcuate line. Below this level, fat surrounding inferior epigastric vessels can be pushed cranially towards rectus abdominis. A dissection plane can be developed between this fat and the perivesical fat, down into the Cave of Retzius. Pushing the bladder posteriorly reveals the pubic symphysis anteriorly, pubic tubercles bilaterally, and the femoral rings with the accompanying inguinal, lacunar, and pectineal (Cooper's) ligaments. In 10% of cadavers, the corona mortis lies between the obturator artery and inferior epigastrics (or external iliacs). This important landmark can bleed profusely if injured. Dissection is then continued laterally into the Space of Bogros, to reveal the myopectineal orifice (Figure 4). The authors advise further dissection, out to the anterior superior iliac spine and beyond; developing this space will facilitate bottom‐up TAR. During dissection, the genitofemoral nerve can be identified on the psoas body, the femoral nerve lies lateral to the psoas, and the lateral cutaneous nerve of the thigh lies on the pelvic side wall, accompanied by its vessel. The anterior femoral cutaneous nerve is a branch of the femoral nerve and can be challenging to identify. All nerves lie in the “triangle of pain” and care must be taken to avoid injury.

### 4.3 Cranial Dissection

Once caudal dissection is complete, the retrorectus space can be extended cranially. However, lateral extension tends to be more constrained by neurovascular bundles that course more medially, risking injury (Figure 1A). In the midline, the fatty rhomboid of the trident becomes visible, which signals the point at which preperitoneal fat should be dissected under the posterior rectus sheath to gain access to the preperitoneal space. As discussed previously, the rectus sheath complex inserts anterior to xiphisternum and costal margin, so entering the subxiphoid preperitoneal space requires division of the linea alba up to the xiphoid, and division of both posterior sheaths (at the point where they insert into the xiphoid and the costal margin). This opens the plane between the peri‐xiphoid fat (fatty ball) and preperitoneal fat (fatty rhomboid). Peri‐xiphoid fat should be pushed anteriorly and preperitoneal “fatty rhomboid” posteriorly (Figure 3C,D).

### 4.4 Classic Top‐Down TAR

**FIGURE 5:**
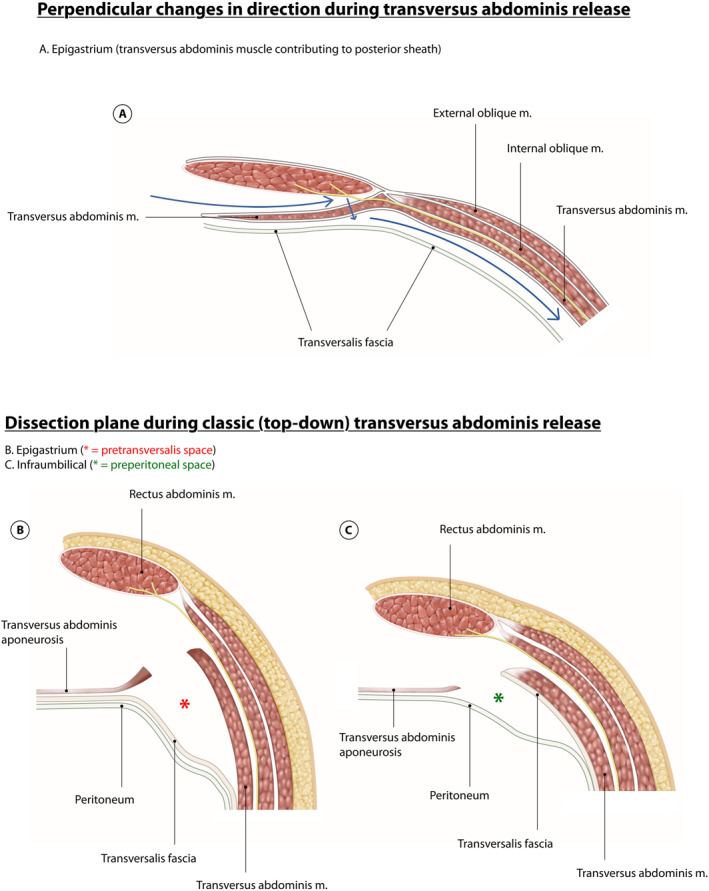
(A) Classic top‐down TAR requires two deliberate perpendicular changes in direction of dissection. The first change in direction occurs from lateral (retrorectus) to posterior when cutting through transversus abdominis. (B) The second change in direction occurs from posterior (once through transversus abdominis) to lateral again, with dissection continuing in the pre‐transversalis plane. (C) As TAR proceeds in caudal direction, there is a subtle change of plane from pre‐transversalis plane to preperitoneal plane. This is because dissection is through the muscle cranially and aponeurosis caudally as the anatomical semilunar line moves in an inferolateral direction.

**FIGURE 6:**
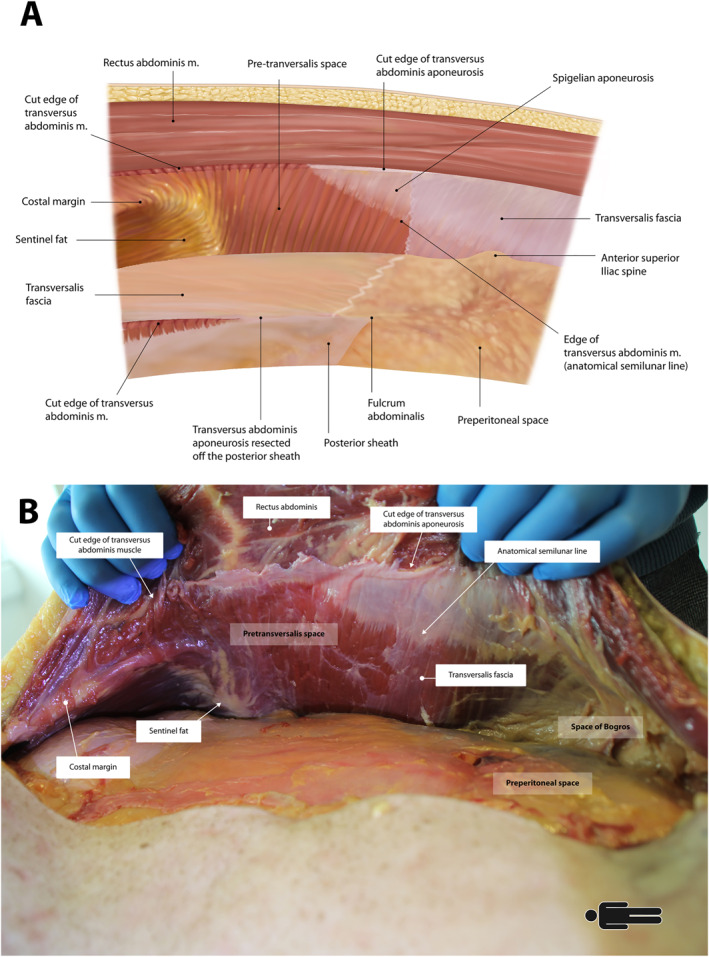
(A) Computerized illustration of the preperitoneal and pre‐transversalis spaces after left‐sided TAR. (B) Cadaveric photograph after left sided TAR. Note the transition point of transversalis fascia on transversus abdominis muscle. A wide space is available for extraperitoneal mesh placement extending round to quadratus lumborum.

In the epigastrium the transversus abdominis and posterior lamella of the internal oblique insert medially into the posterior sheath. To perform a top‐down TAR, the fascia of the posterior lamella of internal oblique (medial to the lamppost) is incised to reveal transversus abdominis. Using Lahey's forceps, small amounts of this muscle are cut progressively to uncover the posterior sheath. This requires a conscious change of direction from lateral dissection in the retrorectus plane to dissection perpendicularly down (aiming posteriorly) when cutting through transversus muscle (Figure 5A). Dissection progresses caudally, parallel to the lateral border of posterior sheath (ambivium), to reach the arcuate line. Because the medial border of transversus moves laterally as dissection proceeds caudally, the tissue being divided changes from transversus muscle to transversus aponeurosis (Figure 5B). Once the retromuscular space under transversus abdominis is opened, another perpendicular change of direction is required, allowing extension further laterally in the pre‐transversalis fascia plane (Figure 6). Extending the dissection posterolaterally reveals psoas and quadratus lumborum. Cranially, dissection continues in the pre‐transversalis plane to reach the sentinel fat at the costal margin, and beyond that into the pre‐diaphragmatic fascia plane (Figure 3). Continuing further, and in a medial direction, reveals the central diaphragmatic tendon. At this point, care must be taken to follow the diaphragmatic curve exactly, so as to avoid iatrogenic Morgagni hernia via inadvertent incision. It is difficult to perform TAR without peritoneal breach. If this occurs, the dissection plane should be changed and continued rather than immediate repair attempted. Peritoneal defects can be repaired subsequently via suture or by omental patch. Recall that the correct plane can be identified by having transversus abdominis muscle “up” and the peritoneum “down.” If dissection occurs between two layers of muscle, either the interoblique plane (between external and internal oblique) or the retro‐oblique plane (between internal oblique and transversus abdominis) has been entered, and the dissection course should be corrected.

### 4.5 Madrid PCS

This technique was first published in 2018 by Robin‐Lersundi and colleagues [28] and revolutionized how TAR is performed. The technique is meticulous at preserving muscles, nerves, and blood vessels, and ultimately abdominal wall function. Dissection begins at the arcuate line and proceeds cranially. At the fulcrum abdominalis, a finger should be placed underneath the inferior edge of posterior sheath (or arcuate line), and is cushioned by preperitoneal fat at the root of the fatty trident. Following this, the posterior rectus sheath/transversus abdominis aponeurosis is cut medial to the neurovascular bundles, in a cranial direction (Figure 2C). This aponeurotic layer should be divided at its most medial limit to avoid dividing transversus abdominis muscle and to preserve neurovascular bundles that insert more medially into rectus abdominis as the dissection continues.

AWR surgeons must understand that as a bottom‐up TAR progresses cranially, the extraperitoneal space of dissection lateral to the ambivium changes from preperitoneal to pre‐transversalis space (Figure 6). This is because there is usually no preperitoneal fat between the lateral and medial prongs of the fatty trident. This frustrates preperitoneal plane dissection, so transferring to the pre‐transversalis space creates an extra layer of protection, facilitating peritoneal preservation. A similar transition, from pre‐transversalis to preperitoneal, occurs with classic top‐down TAR, but this occurs by default, not needing the conscious plane change required for bottom‐up TAR.

It is also vital that with bottom‐up TAR, retrorectus dissection should stop approximately 6 cm below the xiphisternum (Figure 2C). Doing so avoids damaging epigastric neurovascular bundles which extend medially, and fibers of the pars abdominalis. Instead, preperitoneal fat both sides of the midline are entered beneath the posterior rectus sheaths to access the preperitoneal space. The plane between peri‐xiphoid fat (fatty‐ball) and the preperitoneal fatty rhomboid must be developed. Progressing laterally, dissection in the preperitoneal plane is almost impossible because fat from the midline prong tapers, and dissection must move anteriorly into the pre‐transversalis plane to avoid peritoneum. The pre‐transversalis plane is then extended both cranially and caudally. Caudally it adjoins the pre‐transversalis plane created previously by the bottom‐up TAR, and once both pre‐transversalis spaces are connected, the posterior sheath is suspended as a single band of fascia. Dividing this creates a continuous extraperitoneal plane for mesh placement (Figures 3B and 6). For M1 hernias [20], which are rare and difficult to repair, this subxiphoid dissection is imperative to achieve adequate mesh overlap.

## 5 Conclusion

In summary, as complex abdominal wall reconstruction continues to advance, a comprehensive understanding of abdominal wall anatomy becomes increasingly essential. This expert consensus synthesizes the key anatomical and operative principles and translates them into a structured framework for teaching PCS. This article is intended as a reference guide for surgical training.

## Author Contributions

**Lawrence Nip:** writing – original draft, writing – review and editing, data curation, formal analysis, visualization. **Samuel G. Parker:** conceptualization, writing – review and editing, methodology, validation, formal analysis, resources. **Andrea Scala:** conceptualization, writing – review and editing, methodology, validation, resources. **Steve Halligan:** conceptualization, writing – review and editing, methodology, validation, supervision. **Timothy A. Rockall:** conceptualization, writing – review and editing, methodology, validation, resources. **Rhys Thomas:** conceptualization, writing – review and editing, methodology, validation, resources. **Pasquale Giordano:** conceptualization, writing – review and editing, methodology, validation, resources. **Gregory Simpson:** conceptualization, writing – review and editing, methodology, validation, resources. **Praminthra Chitsabesan:** conceptualization, writing – review and editing, methodology, validation, resources. **Marja Boermeester:** conceptualization, writing – review and editing, methodology, validation, resources. **Sarah Zhao:** conceptualization, writing – review and editing, methodology. **Alastair C. J. Windsor:** conceptualization, writing – review and editing, methodology, validation, resources. **Miguel Angel Garcia‐Urena:** conceptualization, writing – review and editing, methodology, validation, resources, supervision.

## Funding

The cadaveric course and manuscript fees were funded by the TELA Bio. The funder did not have any role in the study design, thematic analysis, interpretation of data, writing the report or decision to submit for publication.

## Ethics Statement

This research was conducted in accordance with the Human Tissue Act 2004 and the institution held appropriate license for anatomical examination of cadavers. Research Ethics Committee approval was not required for this research.

## Consent

All individuals whose cadavers are presented in this manuscript provided informed consent for publication prior to death, specifically for the purpose of medical education.

## Conflicts of Interest

The authors declare no conflicts of interest.
